# Degenerative Gastrocnemius Muscle Changes in a Goat Tibial Ostectomy Model Persist 10 Months After Splint Removal

**DOI:** 10.3390/muscles5010020

**Published:** 2026-03-04

**Authors:** Benjamin T. Baker, Rebecca E. Rifkin, Becka Klein, Brittani Lopez, Remigiusz M. Grzeskowiak, Elizabeth Croy, Xiaojuan Zhu, Pierre-Yves Mulon, David E. Anderson, Dustin L. Crouch

**Affiliations:** 1 Department of Biomedical Engineering, Tickle College of Engineering, University of Tennessee, Knoxville, TN 37996, USA; 2 Department of Large Animal Clinical Sciences, College of Veterinary Medicine, University of Tennessee, Knoxville, TN 37996, USA; 3 Office of Innovative Technologies, University of Tennessee, Knoxville, TN 37996, USA

**Keywords:** coaptation, atrophy, musculoskeletal, animal, disuse, recovery

## Abstract

Major orthopedic limb surgery is often accompanied by external coaptation; the combined effect of these interventions can lead to muscle atrophy and functional impairment. Large animal models, including goats, are commonly used to study orthopedic interventions, yet longitudinal data on muscle changes after such interventions are limited. This study quantified gastrocnemius muscle adaptations in adult Boer-cross goats undergoing a clinically representative unilateral tibial segmental ostectomy and external coaptation protocol. Muscles on the operated side exhibited statistically significant decreases in mass, length, optimal fiber length, and CSA, and increases in nucleus density compared to muscles on the contralateral, non-operated side (*p* < 0.05). Although muscle properties showed partial recovery over time, mass and CSA remained 20–30% lower on the operated side than on the non-operated side at 12 months post-surgery despite cast removal at about 2 months post-surgery. Muscle CSA was positively correlated with bone mineral density and peak vertical ground reaction forces measured during the in vivo study. The extent of muscle recovery in the goat model was less than that observed for other mammalian models of hindlimb remobilization. More research is needed to understand the complex interaction between surgery, external coaptation, and muscle properties in the goat model.

## 1. Introduction

External coaptation (e.g., splints and casts) is commonly prescribed for many orthopedic conditions [1,2]. The goal of external coaptation is to restrict motion and mechanical loading of injured and immature repair tissues to facilitate healing and prevent reinjury. However, there are recognized and documented adverse effects on muscle structure and function resulting from the use of external coaptation. For example, external coaptation reduces the loading of skeletal muscle–tendon units that traverse the coapted and adjacent joints, which can lead to muscle atrophy and fibrosis [3]. When a muscle is maintained in a shortened length, it may undergo architectural remodeling, characterized by the loss of sarcomeres in series, which reduces the optimal fiber length [4]. Muscle atrophy, fibrosis, and shortening can impair strength, mobility, and range of motion of the joint(s) crossed by the affected muscles, resulting in functional impairment [5,6]. Decreased strength and range of motion cause patients to adopt compensatory movements [7] that can reduce the energetic efficiency of movement [8,9] and lead to secondary musculoskeletal conditions such as pain and overuse injuries [10]. The removal of external coaptation allows movement and muscle loading to resume, which is expected to lead to the recovery of muscle structure and function [11].

In the context of major orthopedic reconstruction, the mechanical environment of the limb is altered by a complex interplay of clinical factors. Although external coaptation, such as casting or splinting, is a known driver of disuse atrophy, it rarely acts in isolation in clinical scenarios. Other typical factors include surgical trauma to the bone and soft tissues, post-operative inflammation, and the psychological and physiological impacts of pain [12,13,14]. These collective factors can lead to sustained periods of attenuated weight-bearing and reduced range of motion, which together could affect the rate and extent of muscle degeneration and recovery. Clinically representative models are needed to fully elucidate the complex interactions between these multifactorial stressors and their collective impact on long-term musculoskeletal health.

Several large animal species, including goats, pigs, and sheep, are commonly used to test and quantify the effects of orthopedic interventions [15,16,17,18,19,20,21,22,23,24]. Large animal models are valuable for orthopedic research because of their similarities to humans with respect to biomechanical factors such as limb scale, bone structure, and load magnitude [25,26,27,28]. One specific model, the tibia segmental defect model, has been used in goats [29,30,31,32,33] and sheep [34] to study methods to improve fracture healing in long bones. In this model, orthopedic implants, such as plates or intramedullary nails, are used for direct stabilization of the bone after a segment has been removed. External coaptation is often used in conjunction with orthopedic implants to further stabilize and protect the operating site. However, despite the common use of large animals in orthopedic research, longitudinal studies of skeletal muscle following orthopedic procedures that involve external coaptation are limited. This gap limits the translation of findings in large animal models to clinical applications in human patients.

The goal of our study was to quantify changes in gastrocnemius muscle properties in goats that underwent unilateral tibial ostectomy and plating, followed by two months of hindlimb external coaptation. We hypothesized that the multi-factorial orthopedic intervention would have transient effects on the gastrocnemius muscles without similar effect on the contralateral, non-operated side. To test the hypothesis, we assessed within-limb and between-limb muscle differences and correlated these changes to in vivo measurements of select gait biomechanics and bone healing variables. The results of this study contribute new information about the goat model that will be useful for planning future pre-clinical models of orthopedic interventions. Furthermore, the data could inform the design of clinical treatments to mitigate muscle changes.

## 2. Materials and Methods

**Figure 1:**
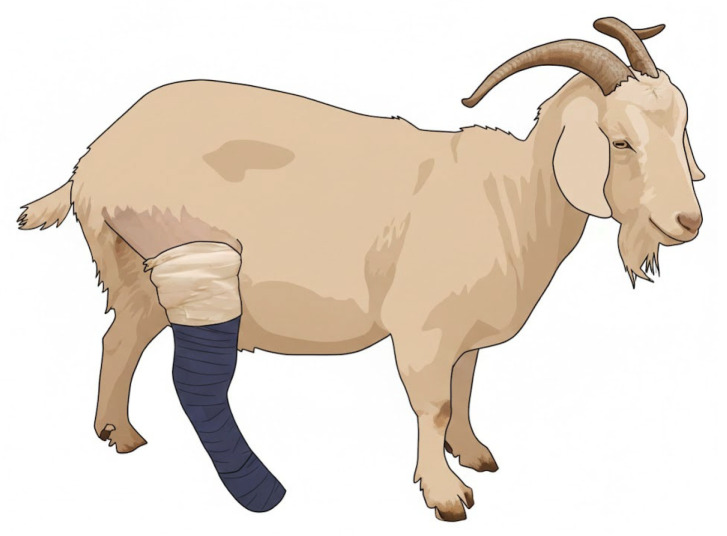
Illustration of the external coaptation that was applied to the right, operated hindlimbs using a Robert Jones splint bandage.

All in vivo animal procedures were approved by the Institutional Animal Care and Use Committee at the University of Tennessee (protocol 2383). As part of a larger study of a regenerative bone scaffold [35], adult Boer-cross goats were divided into three groups: control (c), experimental scaffold(s), and a scaffold with *E. coli*-derived BMP2 growth factor (sBMP2). During the larger study, each goat in all groups underwent mid-diaphyseal segmental ostectomy of the right tibia, followed by stabilization using a custom locking plate. After surgery, external coaptation was applied to the right, operated hindlimb using a Robert Jones splint bandage (Rear Leg/Tarsal Quick Splint, Large, Jorgensen Laboratories, Loveland, CO, USA), while the left side was unaffected and served as a contralateral limb control. The splints, which spanned the limb from the toe to the level immediately below the femorotibial joint (Figure 1), were changed regularly and finally removed approximately 2 months after surgery.

At pre-determined timepoints (3-, 6-, 9-, and 12-months after surgery), the goats were euthanized, and the tibia was harvested from the operated limbs. During the collection of initial specimens at the 3-month time point, we observed obvious qualitative differences in gastrocnemius muscle size between the operated and non-operated hindlimbs. At that time, we devised this study to prospectively harvest the gastrocnemius muscles from the remaining animals (6-, 9-, 12-month groups) for quantitative assessment. Although we did not capture gastrocnemius muscle data from all animals in the original study, a minimum of 3 animals per group–timepoint combination were included in an analysis of muscle mass and length. More extensive measurements of muscle morphology, as described below, were made on a subset of the gastrocnemius muscle specimens.

**Figure 2:**
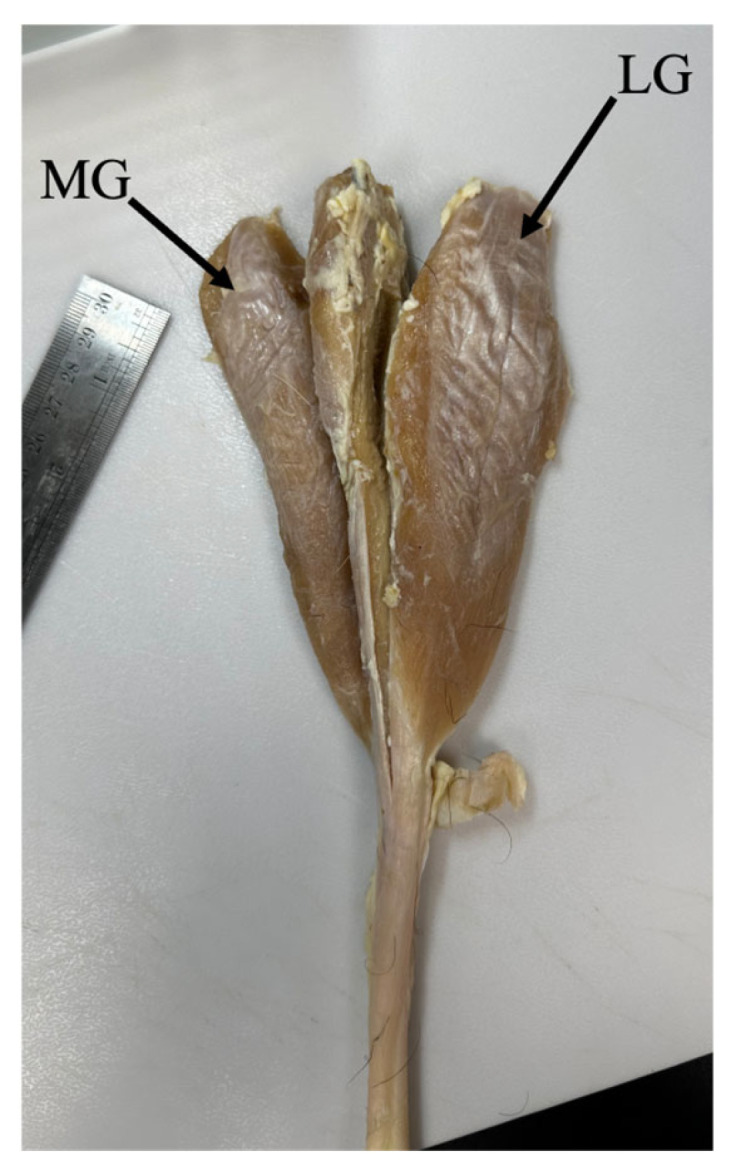
Triceps surae muscle–tendon unit from the right hindlimb of one goat. The lateral gastrocnemius (LG) and medial gastrocnemius (MG) were separated from the tendon at the muscle-tendon junction for further analysis.

The triceps surae muscle–tendon unit (lateral gastrocnemius (LG), medial gastrocnemius (MG), and soleus muscle; Figure 2) was harvested from the right (operated) and left (non-operated) hindlimb and was fixed in 10% neutral-buffered formalin for at least 2 weeks. Then, the lateral gastrocnemius (LG) and medial gastrocnemius (MG) muscles were dissected, with the tendon removed at the muscle–tendon junction. The LG and MG were stored in ethanol for at least one week prior to the measurement of muscle properties.

From the LG and MG, muscle mass was measured ($m_{m}$) using a digital scale; muscle length ($l_{m}$) and muscle fiber length ($l_{f}$) were measured using a digital caliper. For each muscle specimen, each of the three variables ($m_{m}$, $l_{m}$, and $l_{f}$) were measured three times. Then, the average of the three repeat-measured values was calculated.

**Figure 3:**
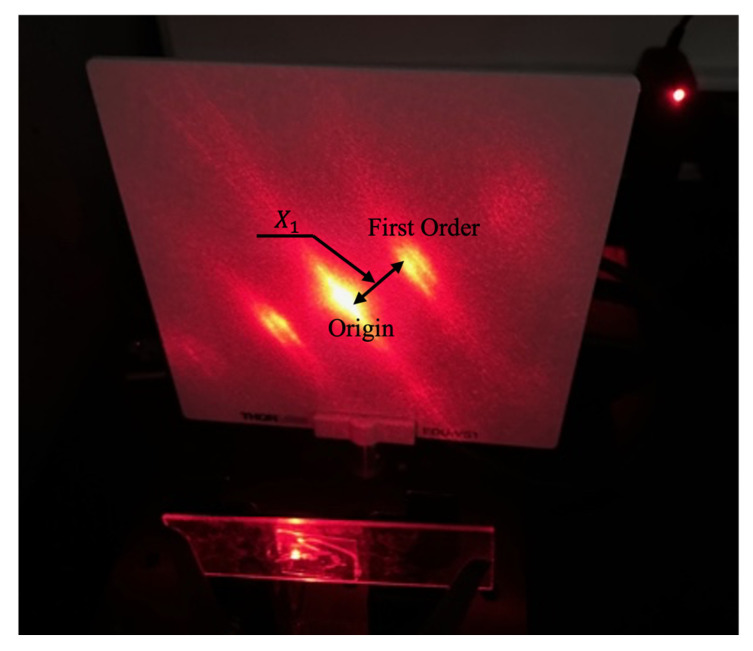
Projection of the laser diffraction pattern. Diffraction length was measured from the origin to the first order diffraction.

Sarcomere length $\left(l_{s}\right)$, was measured using a conventional laser diffraction [36] method. Muscle fiber bundles approximately 1 cm long were dissected from the mid-section of each muscle and placed on a glass slide. Individual fibers were teased apart and covered with a glass cover slip. The slide was mounted on a stand. A laser beam generated by a He-Ne laser (HNLS008L, Thorlabs, Inc., Newton, NJ, USA) was directed through the fibers, and the resulting diffracted light was projected onto a white panel. The diffraction length (X_1_), the distance from the origin to the first-order band, was measured with a digital caliper (Figure 3).

The general formula for computing sarcomere length from the diffraction length is as follows:

(1) $$l_{s}=\frac{n\lambda }{\sin\left(\tan^{-1}\left(\frac{X_{n}}{h}\right)\right)}$$

In Equation (1), n is the diffraction band order, which was equal to one; the wavelength (λ) of the laser was 633 nm; and h is the distance from the glass slide to the projection screen, which was 0.0936 m.

Optimal fiber length $\left(l_{f}^{0}\right)$ was calculated using the following equation:

(2) $$l_{f}^{0}=l_{s}^{0}\left(\frac{l_{f}}{l_{s}}\right)$$

In Equation (2), $l_{s}^{0}$ was set to 2.2 µm, the reported optimal sarcomere length for mammalian skeletal muscle [37].

The cross-sectional area (CSA) of each muscle was calculated as follows:

(3) $$CSA=\frac{m_{m}}{l_{f}^{0}\rho_{m}}$$

Equation (3) is adapted from the typical equation for physiologic cross-sectional area (PCSA), which also accounts for pennation angle [37,38]. The density of skeletal muscle $\left(\rho_{m}\right)$ was assumed to be 1054 $\frac{kg}{m^{3}}$, as reported in the literature [37].

For a subset of animals at each timepoint (n = 8 at 6 months, n = 1 at 9 months, n = 7 at 12 months), muscle tissue samples were dissected from the mid-section and cut to approximately 1 mm thickness, approximately perpendicular to the muscle fibers, using a surgical scalpel in preparation for histology sectioning. The samples were placed in cassettes with one cut side facing down to ensure that histology sections were prepared perpendicular to the long axis of the muscle fibers. The histology sections were stained with hematoxylin and eosin (H&E) stain. For each section, the number of nuclei per high-powered field (HPF, 40× magnification) was counted manually using a standard compound light microscope (M82, OMAX, Sejong-si, Republic of Korea) and was reported as the nucleus density (ND). For one histology section per muscle, we measured ND in five separate HPF regions, then averaged the ND values across regions.

In the larger in vivo study [35], we measured and reported the bone mineral density (BMD) and peak vertical ground reaction force (vGRF) during the weight-bearing phase of stride for all goats. We utilized these data to create a subset that included only (1) the operated limb, (2) the study duration endpoint for each animal, and (3) the animals from which we measured the muscle properties for the current study. The data subset was included in the analysis described below.

All variables were tested for normality using the Shapiro–Wilk test and QQ plot. All variables met the normality assumptions. A mixed-model ANOVA with a nested factor split plot design was used for the data analysis, because time points were nested within the three treatment groups (not all groups contained all timepoints), to determine if there was a significant effect of group, limb side, the timepoint nested within the group (denoted as time[group]), and their interaction (group by side and side by time[group]) with group and time[group] as a whole plot factor, side (operated and non-operated) as a split plot factor and each individual goat as a random factor. The analysis was conducted both for each muscle separately (LG and MG) and the muscles combined (LG + MG).

The BMD and vGRF were assessed using two-way ANOVA to test the effect of group and time, and time by group interaction. Least squares means were computed and separated using the least significant difference (LSD) correction method. A Shapiro–Wilk W and QQ normality plots were used to evaluate the normality of ANOVA residuals. All statistical assumptions regarding normality and equality of variances were met.

A Pearson correlation analysis was performed between select muscle properties (muscle mass, muscle length, optimal fiber length, and CSA) and previously reported bone (BMD) and gait function (peak vGRF) outcomes. For all statistical comparisons, *p*-values < 0.05 were considered significant. JMP Pro Version 17 was used for all the analyses (JMP Statistical Discovery LLC, Cary, NC, USA).

## 3. Results

**Figure 4:**
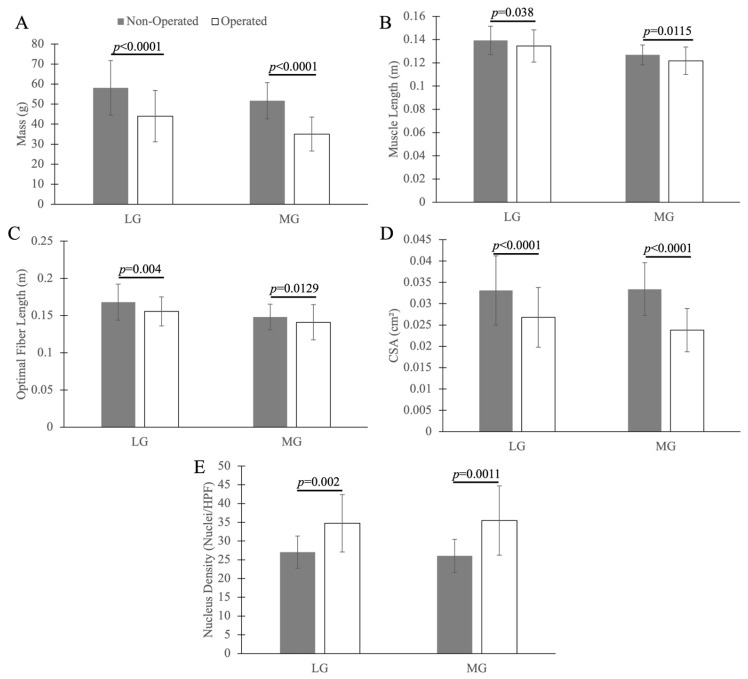
ANOVA comparison of the muscle properties of the lateral gastrocnemius (LG) and medial gastrocnemius (MG). Muscle properties include muscle mass (**A**), muscle length (**B**), optimal fiber length (**C**), cross-sectional area (CSA) (**D**), and nucleus density (ND) (**E**). The error bars represent ±1 standard deviation.

Side was the only significant factor for mass, length, optimal fiber length, CSA, and nucleus density (*p* < 0.0001, *p* = 0.038, *p* = 0.004, *p* < 0.0001, and *p* = 0.002 for LG and *p* < 0.0001, *p* = 0.012, *p* = 0.013, *p* < 0.0001, and *p* = 0.001 for MG, respectively, as compared to the contralateral limb; Figure 4). Group, time[group], and their interaction were not significant (*p* > 0.05) for mass, length, optimal fiber length, or CSA. Mass, length, optimal fiber length, and CSA were less on the operated side than on the non-operated side. Conversely, the nucleus density was greater on the operated side than on the non-operated side.

**Figure 5:**
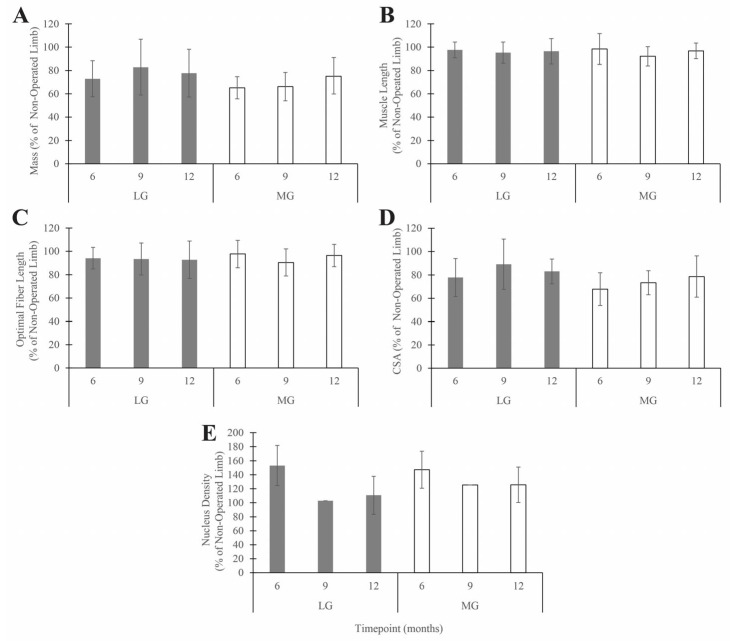
Muscle property values measured from the lateral gastrocnemius (LG) and medial gastrocnemius (MG) muscles in the operated limb, averaged across animals and groups at each timepoint and expressed as a percentage of the non-operated limb value. Muscle properties include (**A**) muscle mass, (**B**) muscle length, (**C**) optimal fiber length, (**D**) cross-sectional area (CSA), and (**E**) nucleus density. The error bars represent ±1 standard deviation. There was no statistically significant effect of time or muscle for any muscle property (*p* > 0.05).

Though the effect of timepoint was not statistically significant (*p* > 0.05), there was a gradual trend of recovery of muscle mass and CSA over time (Figure 5). In any case, even at the 12-month timepoint, which was about 10 months after the splints were removed, muscle mass and CSA were about 20–30% lower, on average, on the operated side than on the non-operated side.

**Figure 6:**
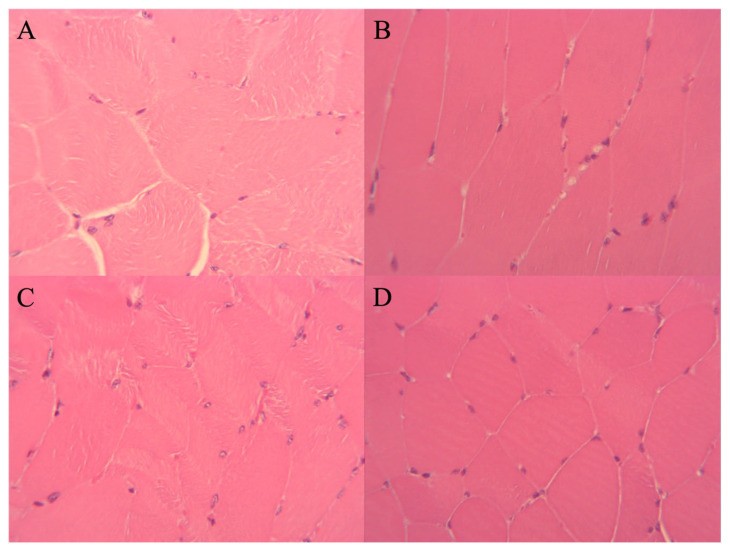
Representative histology sections from an animal in the control group at the 12-month timepoint. (**A**) Lateral and (**B**) medial gastrocnemius on the non-operated side. (**C**) Lateral and (**D**) medial gastrocnemius on the operated side (high-powered field (HPF), 40× magnification).

From representative muscle histology sections (Figure 6), at the 12-week timepoint, the muscle fiber diameters and cross-sectional areas appeared smaller. Qualitatively, there were more nuclei on the operated side than on the non-operated side. These qualitative observations are consistent with the quantitative between-side differences in nucleus density, as shown in Figure 3.

**Figure 7:**
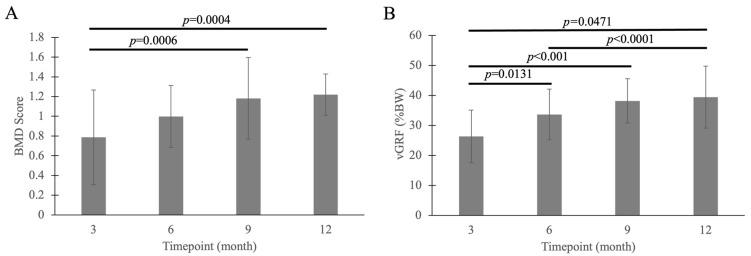
There was a significant effect of timepoint for both BMD (**A**) and peak vGRF (**B**), with both increasing from month 3 to month 12. The grey bars represent the mean, and the error bars represent ±1 standard deviation.

Timepoint had a significant effect on BMD (*p* = 0.001) and peak vGRF (*p* < 0.0001) (Figure 7). Both BMD and peak vGRF increased over time during the measurement. Month 3 had lower BMD compared with months 9 and 12 (*p* = 0.001 and *p* = 0.001, respectively). Peak vGRF was lower at month 3 compared with months 6, 9, and 12 (*p* = 0.013, *p* < 0.001, and *p* < 0.0001, respectively), and lower at month 6 compared with month 12 (*p* = 0.047). No significant effect was observed for either group or group by time interactions.

**Figure 8:**
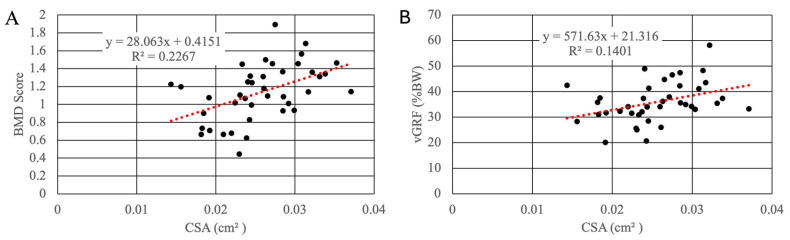
Correlation between BMD and CSA (**A**) and between peak vGRF and CSA (**B**).

BMD was significantly positively correlated with muscle mass on the operated (r = 0.497, *p* = 0.001) and non-operated (r = 0.35, *p* = 0.027) side. Likewise, vGRF was significantly positively correlated with muscle mass on the operated (r = 0.477, *p* = 0.002) and non-operated (r = 0.365, *p* = 0.022) side. CSA was significantly positively correlated with BMD (r = 0.48, *p* = 0.002) and vGRF (r = 0.37, *p* = 0.019) on the operated side only (Figure 8).

## 4. Discussion

The most predominant between-side differences we observed were less muscle mass and cross-sectional area on the operated side compared to the non-operated side. Muscle optimal fiber length was also lower on the operated side, but the between-side difference was not as large as for mass and cross-sectional area. The muscle deficits in the operated limb likely reflect the combined impact of several factors inherent to this major orthopedic reconstruction model. First, surgical trauma to the bone and surrounding soft tissues, combined with the resultant post-operative pain and discomfort, likely contributed to a reduction in spontaneous limb use and weight-bearing. Furthermore, the application of external coaptation for 2 months post-surgery effectively restricted joint motion, thereby significantly reducing the mechanical loading and length changes typically experienced by the gastrocnemius muscle–tendon units. This sustained period of attenuated biomechanical input, where the muscle is maintained in a relatively static, underloaded state, is a well-documented catalyst for rapid proteolysis and a subsequent decrease in muscle mass [11,39,40]. Consequently, the observed changes should be viewed as a response to a multifactorial clinical scenario involving the initial surgical insult, the inflammatory response, and the secondary effects of prolonged external coaptation.

External coaptation is one mechanism used to immobilize joints for clinical and research purposes. As noted in the previous paragraph, external coaptation was one of multiple factors that likely contributed to the observed bilateral muscle differences. However, our observations are consistent with those reported previously following a period of joint immobilization in a variety of species. Here we highlight studies involving immobilization of the ankle joint, which is anatomically analogous to the goat’s hock joint, one of the joints spanned by the splint. One study in rats immobilized the ankle joint without any other injury or clinical intervention (e.g., fracture or ostectomy) [39]. The study found that the mass of both the gastrocnemius and soleus muscles were about 40% lower after both 4- and 6-weeks post-immobilization than in a non-immobilized control group; conversely, the number of sarcomeres in series along a muscle fiber (a direct correlate of optimal fiber length) was only about 6% less on the immobilized side, and this difference was not statistically significant for all conditions. In 18 adult human patients, after 6 weeks of ankle cast immobilization due to an ankle fracture, the cross-sectional area of the LG and MG muscles were less on the immobilized side by 17.1% and 23.3%, respectively, compared to the non-immobilized side [40]. Another study in humans with ankle (malleolus) fractures reported a ~25% decrease in gastrocnemius muscle CSA by 7 weeks after ankle cast immobilization [11]. Thus, although distinct inter-species differences in biomechanics, anatomy, and other factors must be acknowledged, our results and those of previous studies demonstrate a consistent biological response: joint immobilization is strongly associated with significant muscle atrophy [3].

One other notable bilateral difference we observed was a greater nucleus density (ND) on the operated side compared to the non-operated side. There are several factors that may have contributed to a higher nucleus density in the operated limb than in the non-operated limb. One factor may have been that, because the muscles were atrophied (i.e., smaller mass and CSA), the muscle fibers likely had a smaller diameter and cross-sectional area [41,42]. Previous studies report that the number of nuclei in muscle fibers remains constant even with substantial atrophy [43,44]. If the fiber cross-sectional area decreases and the number of nuclei is preserved, then the nucleus density as measured in our study would increase. A second factor was that the number of nuclei in each muscle fiber may have increased during the hypertrophic recovery phase [45]. Finally, there was likely an infiltration of non-muscle support cells, such as immune cells and muscle satellite cells [46,47,48,49,50,51], among atrophied and recovering muscle fibers that would increase the number of nuclei in a given area.

There was a trend of recovery (i.e., less bilateral difference over time) of muscle mass and CSA, though the recovery was incomplete. Other studies have reported near-complete recovery of muscle CSA after cast/splint removal and joint remobilization. For example, one human study reporting a ~25% decrease in gastrocnemius muscle CSA after 7 weeks of ankle cast immobilization also observed that CSA increased by 20–30% 10 weeks after the cast was removed [11]. In another study, healthy adult male human subjects underwent ankle cast immobilization for 2 weeks; the 6% loss in muscle CSA at the cast removal timepoint was almost completely recovered 2 weeks post-removal, though ankle plantarflexion strength remained 9% lower than normal [52]. Following 3 months of hindlimb ankle cast immobilization in rats, soleus muscle mass increased by over 50% to near-normal 14 days after cast removal [53]. It is unclear whether hindlimb loading was a factor, since the peak vertical force on the operated hindlimb during walking (Figure 6) was normal (about 35% BW [35]) by the 9-month timepoint. Therefore, more research is needed to explain the incomplete muscle recovery in our goat model.

Though hindlimb loading appeared normal by the 9-month timepoint, chronically reduced biomechanical loading on the operated limb could potentially explain, at least partly, the persistent muscle deficits. This hypothesis is supported by our findings that muscle mass and CSA were significantly positively correlated with both BMD and vGRF on the operated side. Such a hypothesis is consistent with current scientific understanding of how mechanical loading affects muscle and bone. vGRF is an indirect indicator of potential load magnitude in (1) the regenerative bone scaffold in the tibia in which BMD was measured and (2) the hindlimb muscles. Greater limb loading is associated with increases in bone mineral density [54,55] and muscle mass [56], whereas reduced loading has the opposite effect [57]. In a subsequent study by our team using a very similar goat ostectomy model, peak vGRF remained significantly lower than in the non-operated hindlimb at 6, 9, and 12-months post-surgery [58].

Another potential factor that may explain the differences in muscle recovery reported by previous studies [11,52,53] and our study is a difference in walking gait biomechanics among species. The previous studies reported muscle recovery in rats [53] and humans [11,52], which are species that walk with a plantigrade (i.e., walking on the soles of the feet) gait. Conversely, goats are unguligrade, walking on their toes that are protected by hooves. Because of these gait differences, the goat ankle joint is more extended (i.e., plantarflexed), ranging from 140–160° during the stance phase [59]; conversely, the human ankle angle ranges from about 10° of dorsiflexion to 20° of plantarflexion during stance [60]. The more extended ankle joint posture in goats may suggest that they have relatively less ankle plantarflexion moments and, therefore, less loading of the ankle plantarflexor muscles (e.g., gastrocnemius and soleus) during walking. However, the normalized peak plantarflexion moment is actually higher in goats (~2.5 N m kg^−1^) than in humans (less than 2.0 N m kg^−1^). A more extensive literature review of inter-species differences in biomechanics, anatomy, and muscle plasticity is needed to better understand the extent to which they may contribute to differences in post-immobilization muscle recovery. Furthermore, given that there are many inter-species differences that could influence muscle recovery, more extensive, controlled, multi-factorial studies are needed to better understand factors that contribute to muscle recovery, specifically in goats.

Other factors besides biomechanical loading may have contributed substantially to persistent muscle deficits. This is suggested by our finding that, though mean values of peak vGRF increased by 50% (*p* = 0.047) from the 3- to 12-month timepoint (Figure 7B), there was no significant effect of timepoint for any muscle property. One potential factor is mild pain and discomfort with use of the operated limb due to incomplete bone healing or interaction between the bone and fixation plate; such pain and discomfort could have been overlooked if the goats made subtle adaptations in their gait biomechanics to compensate or favor the operated limb. Testing external coaptation separately from surgery would be expected to isolate the effects of pain and discomfort. Additionally, a more comprehensive biomechanical dataset of ground reaction forces and kinematics would be needed to identify potential compensatory adaptations. Another potential contributing factor is post-immobilization activity and loading. In our study, these were not controlled or monitored. Formal rehabilitation, as would be prescribed for human patients, provides structured activity and loading to the affected joint(s), which would be expected to increase the rate and extent of muscle recovery. Thus, our future studies in goats could test whether increased activity and loading, possibly prescribed through a formal rehabilitation protocol, could increase muscle recovery.

To better understand the relationship between load characteristics and tissue properties in specific cases, more direct measurement of tissue loads is needed. Direct in vivo load measurements are often very challenging or impractical. As practical alternatives to in vivo measurements, computational musculoskeletal models and simulations can be used to calculate muscle and bone loads from experimental biomechanical and anthropomorphic data [61]; some models have been created for large animal models such as sheep [62] and goats [59]. Such models may be useful in future studies to, for example, investigate why gastrocnemius muscle mass remained substantially lower on the operated side compared to the non-operated side in our study.

There are several ways that the muscle assessment could be enhanced in future research. It would be valuable to measure more muscle properties to better understand the effect of hindlimb interventions on muscle structure and function. For example, the pennation angle affects the proportion of muscle fiber force that is generated along the long axis of the muscle–tendon unit. One study of ankle immobilization in humans found that the pennation angle was about 20% higher in the gastrocnemius on the immobilized side compared to the non-immobilized side [40]. Although experimental measurements quantify the extent of muscle changes, mathematical models of muscles [38] can be used to estimate the effect of muscle changes on muscle capacity and behavior. Finally, we only measured the properties of the lateral and medial gastrocnemius, though other muscles cross the ankle joint. The rate and extent of muscle changes due to biomechanical interventions such as joint immobilization are known to vary among muscles.

Some aspects of our study could be improved to increase scientific rigor. First, because this study was started in the middle of another larger study, we did not collect muscle data at the initial 3-month timepoint of the larger study. Additionally, the number of animals removed prematurely due to infection varied among groups. Therefore, the sample size was generally low and not equal among groups. Second, we did not collect “baseline” muscle properties from normal healthy goats; muscle properties in the non-operated limb may not have been normal due to potential functional compensation for the operated limb. Third, as noted above, the observed muscle changes likely reflect the combined effects of surgical trauma, the inflammatory response, and external coaptation; future studies should test these factors independently, if possible, to understand their relative contributions to muscle changes. Despite these limitations in study design, the importance of the goat model and the significant outcomes of our study justify the need for a larger, more rigorous follow-up study.

In summary, our study characterized the longitudinal muscle adaptations following unilateral tibial ostectomy, plating, and post-operative external coaptation in a goat model. We observed significant bilateral differences in muscle mass, cross-sectional area, nucleus density, and, to a lesser extent, optimal fiber length. Muscle deficits on the operated side were consistent with those reported following joint immobilization, though other surgical and post-surgical factors likely contributed. Bilateral muscle differences did not fully resolve within 10 months of cast removal, suggesting that the standard recovery window may be insufficient for restoring pre-surgical muscle health in the goat hindlimb. Muscle properties were positively correlated with bone mineral density and vertical ground reaction forces, suggesting that the persistent deficits in muscle properties may have been due to chronically reduced biomechanical loading in the operated hindlimb. More research is needed to determine the factors that contribute to long-term deficits in muscle properties and biomechanical loading. Additionally, future studies should investigate whether aggressive rehabilitation protocols or pharmacological interventions can mitigate the persistent deficits and improve functional outcomes following complex orthopedic procedures.

## Data Availability

Further inquiries can be directed to the corresponding author.
